# Development and functional evaluation of recombinant type III collagen intrauterine implant gel

**DOI:** 10.1093/rb/rbaf013

**Published:** 2025-03-17

**Authors:** Xinhui Wang, Xiaoju Fan, Yuanxin Zhai, Jie Li, Huilin Sun, Jie Li, Hao Le, Feng Zhang, Li Zhang, Jianhao Wang, Yun Chu, Pengfei Cui

**Affiliations:** School of Pharmacy, Changzhou University, Changzhou 213164, P. R. China; Jiangsu Trautec Medical Technology Co, Ltd, Changzhou 213200, P. R. China; Jiangsu Trautec Medical Technology Co, Ltd, Changzhou 213200, P. R. China; Jiangsu Trautec Medical Technology Co, Ltd, Changzhou 213200, P. R. China; Jiangsu Trautec Medical Technology Co, Ltd, Changzhou 213200, P. R. China; Jiangsu Trautec Medical Technology Co, Ltd, Changzhou 213200, P. R. China; Jiangsu Trautec Medical Technology Co, Ltd, Changzhou 213200, P. R. China; Jiangsu Trautec Medical Technology Co, Ltd, Changzhou 213200, P. R. China; Jiangsu Trautec Medical Technology Co, Ltd, Changzhou 213200, P. R. China; School of Pharmacy, Changzhou University, Changzhou 213164, P. R. China; School of Pharmacy, Changzhou University, Changzhou 213164, P. R. China; Jiangsu Trautec Medical Technology Co, Ltd, Changzhou 213200, P. R. China; School of Pharmacy, Changzhou University, Changzhou 213164, P. R. China

**Keywords:** intrauterine adhesion, rCol III, temperature-responsive hydrogels, endometrial regeneration, anti-fibrosis

## Abstract

Intrauterine adhesion (IUA) is a prevalent complication arising from uterine surgery, significantly impacting women’s fertility and overall quality of life. The conventional clinical approach involves hysteroscopic separation of uterine adhesions, though this method poses operational challenges and carries risks of postoperative re-adhesion. Alternatively, the intraoperative placement of intrauterine devices or support balloons can act as a physical barrier to prevent adhesion formation. However, its effectiveness is limited and it may result in secondary damage to the endothelial tissue. To tackle these challenges, we have engineered a temperature-responsive hydrogel incorporating Pluronic HP407/HP188 pharmaceutical excipients and recombinant type III collagen (rCol III) as a bioactive element. Upon *in situ* injection into the uterine cavity, this hydrogel transitions from a sol–gel phase to a gel in response to body temperature changes, thereby minimizing nonspecific distribution and prolonging the duration of treatment. *In vitro* studies demonstrate that rCol III temperature-responsive hydrogels exhibit favorable biocompatibility, exhibit a recruitment effect on human endometrial stromal cells, suppress the expression of the fibrotic factor transforming growth factor beta 1 and promote angiogenesis. To evaluate its efficacy in preventing IUA via *in vivo* experiments, we employed sexually mature female rats for IUA modeling and compared its performance with a commercially available product, cross-linked sodium hyaluronate gel. The results indicate that rCol III temperature-responsive hydrogels significantly enhance retention at the injury site, substantially promote endometrial regeneration, augment endometrial blood supply and reduce abnormal fibrin deposition. This study suggests that rCol III temperature-responsive hydrogels can effectively prevent post-surgical uterine adhesions, highlighting their potential as a promising adhesion prevention strategy.

## Graphical abstract

**Figure:**
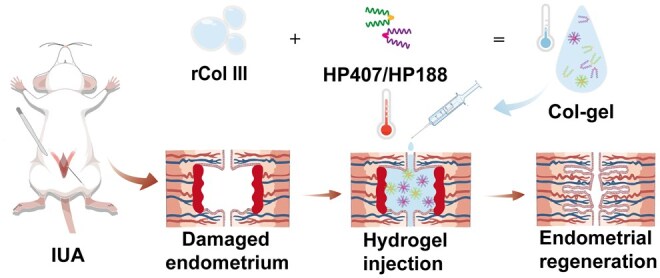

## Introduction

The uterus of a female holds a pivotal role in physiology and reproductive functionality. As an integral aspect of its normal physiological processes, the endometrium possesses the remarkable ability to repair and regenerate itself throughout its lifecycle. During childbirth or menstruation, the uterine lining typically sheds, but scarring is avoided due to the tissue’s rapid and efficient self-repair mechanisms [1]. However, during certain attacks, the endometrial basal layer may be severely compromised by medical procedures or infections. The ramifications are extensive, impeding not only the regeneration of epithelial and mesenchymal cells and the development of neovascularization, but also resulting in an overabundance of fibrin accumulation, among other issues [2, 3]. This condition may disrupt the normal functioning of the endometrium, causing partial or complete blockage of the uterine cavity or (and) the cervical canal, potentially resulting in the formation of intrauterine adhesions (IUA) [4–6].

**Figure 1.**
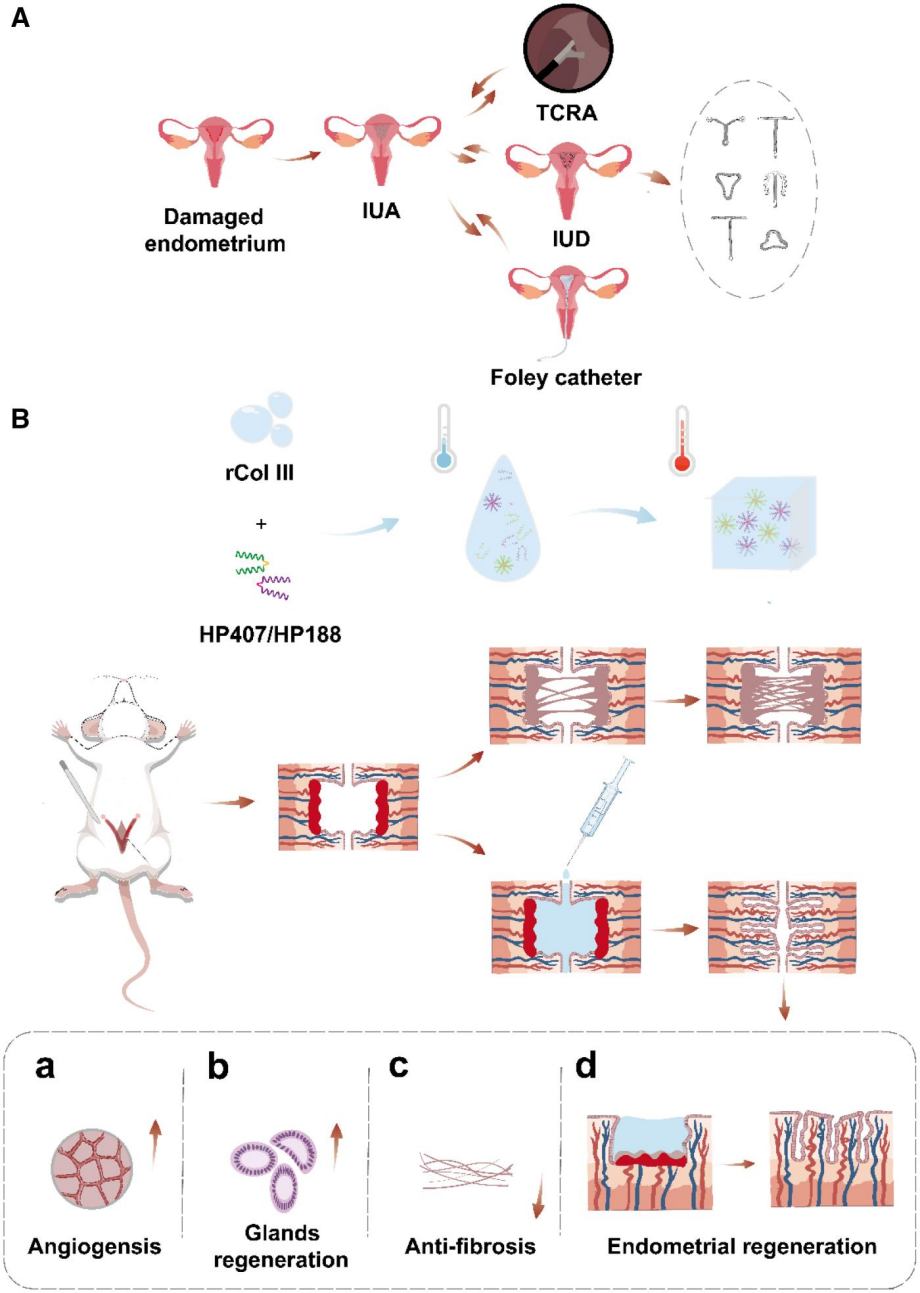
Characteristics of Col-gel and its application in postoperative endometrial repair and uterine adhesion prevention. (**A**) TCRA is a common treatment for IUA; intraoperative intrauterine placement of isolation devices such as birth control devices and support balloons as physical barriers may cause secondary adhesions. (**B**) By injecting Col-gel into the IUA uterus, rCol III is continuously released, promoting neovascularization and gland formation, reducing connective tissue production, facilitating regeneration of the endometrium, and ultimately stopping IUA.

Current technological advancements provide the most effective treatment for IUA, specifically transcervical resection of adhesions (TCRA) [7]. The traditional method of TCRA uses instruments such as dilatation rods, probes and biopsy forceps to isolate the IUA, which is susceptible to uterine perforation, myometrial wall damage and uterine cavity ‘false channel formation’ due to blind operation [8, 9]. Currently, energy intervention is utilized to separate and remove the adherent scar tissue. However, the thermal effect on the tissue inevitably causes damage to the surrounding normal or residual endometrium, thereby elevating the risk of postoperative re-adhesion and scar formation. In addition, intrauterine devices are fairly prevalent as preventive and therapeutic isolation devices. However, these devices act as foreign bodies within the uterus, potentially triggering excessive inflammatory responses. Furthermore, there is a risk of abnormal bleeding, uterine infection, incarceration and uterine perforation [10–12]. Repeated surgeries often fail to achieve satisfactory outcomes and may even further damage the residual endometrial tissue, exacerbate adhesions or induce other diseases. Similarly, the uterine support balloon aims to block trauma through its barrier effect. However, if the balloon’s shape does not align with the uterine cavity, it becomes challenging to fully block the trauma effectively [13]. Additionally, improper control of the fluid or gas injected into the balloon poses a risk of excessive compression of the endometrium, potentially leading to ischemic necrosis and impairing the regenerative repair of the endometrium. The core challenge in managing this condition lies in implementing effective prophylactic measures during the critical repair period of the damaged uterus, aiming to block the adhesion of traumatic surfaces to each other and inhibit the formation of adhesions to a significant extent [14] (Figure 1A).

Several vaginal suppositories [15] and oral medications [16, 17] have been reported as preparations for endometrial repair, yet their efficacy remains poor. This is primarily due to the fact that these existing treatments do not ensure close contact between the therapeutic agent and the site of endometrial injury, nor do they maintain an effective concentration over an extended period. Additionally, the rapid secretion of endometrial mucus for renewal [18, 19] causes a swift loss of therapeutic agents from the lumen of the damaged uterus, impeding the attainment of an effective therapeutic drug concentration. Products such as intrauterine scaffolds and cross-linked hyaluronic acid, despite being used for prophylaxis, face issues of poor compliance and short retention times, thereby hindering the use of current formulations in repairing endometrial lesions. Studies have shown that therapeutic strategies loaded with drugs, stem cells, and exosomes have potential in IUA treatment, but still face problems such as uncontrolled drug release, low stem cell survival, difficulty in exosome extraction, carrier limitations, and individualized differences [9, 20–23]. In addition, the synthesis of some complex drugs may involve multi-step chemical reactions, which are cumbersome and costly; stem cell culture requires specific media, often containing expensive growth factors and serum; and exosome extraction usually requires a large number of cells to be cultured, with demanding cell culture conditions and high raw material costs [24, 25]. Hence, there is an urgent need for a biocompatible agent that offers prolonged retention in the uterus and a robust restorative effect to effectively repair the endometrium.

Type III collagen, a natural substance, constitutes a significant component of reticular fibers within the body. It originates widely from various tissues, including the skin, uterus, intestines and blood vessels. This important protein plays a significant role in regulating essential physiological processes, including wound healing [26]. This protein supports the growth and regeneration of new tissue, enhances tissue integrity and functionality, and is an essential component of the wound healing and tissue repair cycle [27, 28]. Recombinant collagen is synthesized through genetic engineering technology, utilizing recombinant human genes as its foundation. The primary raw material is derived from microbial fermentation and does not involve any animal sources. This ensures safety from the source, eliminates the risk of rejection by the human body, and minimizes viral safety hazards [29]. The main raw material is microbial fermentation, and these microorganisms are cultivated under simple conditions and grow fast enough for large-scale industrial production. In addition, the required raw materials, such as media components (sugars, nitrogen sources, inorganic salts, etc.), are widely available and relatively inexpensive [30]. Recombinant type III collagen (rCol III), a biomaterial renowned for its high bioactivity and biocompatibility, boasts significant clinical applications [31]. It effectively reconstructs the three-dimensional fibrous network of type III collagen, restoring the extracellular matrix environment, sustaining cellular and vascular growth, expediting wound healing, and enhancing the thickness and elasticity of vascular endothelium [32]. The application of rCol III in specific regions has been shown to modulate the disease microenvironment, facilitate repair and regeneration of injured tissues, and hold substantial promise in the management of traumatic conditions [33]. However, rCol III exhibits susceptibility to loss when applied externally, and its efficacy on diseased tissue is short-lived, leading to diminished treatment effectiveness. The research on assisting collagen to work better by combining collagen with hydrogel has provided us with new ideas [34–36].

HP407/HP188 is a poly (ethylene oxide)-poly (propylene oxide)-poly (ethylene oxide) (PEO-PPO-PEO) polymer. Within this polymer, PPO segments undergo dehydration and crosslinking with PEO segments at the phase transition temperature, resulting in the formation of a spherical microgel. Subsequently, these microgels intercrosslink to create a 3D, cross-linked hydrogel [37]. Due to its non-toxicity and good biocompatibility, the US Food and Drug Administration has approved its use as a pharmaceutical excipient. As a temperature-sensitive nonionic surfactant, it naturally forms a gel when it reaches the temperature of the phase transition, forming a protective film on the tissue surface and allowing the active ingredient to remain in contact with the wound surface for a longer period of time, which enhances the therapeutic effect and ensures the efficacy of the treatment [38–40].

In summary, we have developed a temperature-responsive hydrogel loaded with rCol III (Col-gel). This hydrogel remains a free-flowing liquid at room temperature and can be easily injected into the uterine cavity through a syringe, where it forms a gel *in situ* at human physiological temperature. The Col-gel not only extends the residence time of rCol III at the lesion site but also minimizes loss during administration, thereby enhancing efficacy. Additionally, it promotes endometrial repair, reduces abnormal fibrin deposition and significantly inhibits scar formation (Figure 1B). The hydrogel’s preparation process is highly tractable, offering significant potential for the development and application of formulations aimed at preventing uterine adhesions.

## Materials and methods

### Materials

rCol III (purity higher than 98%) was purchased from Jiangsu Chuangjian Medical Technology Co, Poloxamer 407 (HP407) and Poloxamer 188 (HP188) were purchased from BASF SE (Germany). Dulbecco’s Modified Eagle Medium (DMEM) medium, fetal bovine serum (FBS) were purchased from Thermo Fisher Scientific. Low growth factor Matrigel matrix gel was purchased from (Coming-DL Reagent). Recombinant Bovine bFGF and BCA Protein Assay Kits purchased from Beyotime Biotechnology. Human endometrial stromal cells (hESCs) and human umbilical vein endothelial cell (HUVEC) cell lines were purchased from ATCC. Methyl Thiazolyl Tetrazolium (MTT, purity above 98%) was purchased from BioOfroxx (Germany), Dimethyl sulfoxide (DMSO) was purchased from Aladdin Reagent Shanghai Co and endothelial cell culture medium (ECM) was purchased from ScienCell (USA). Transforming growth factor beta 1 (TGF-β1) (21898-1-AP, Proteintech, Rosemont, IL, USA). CY7 dye was purchased from Guangzhou Weihua Biotechnology Co. Cross-linked sodium hyaluronate gel (CLHA) for uterine cavity was purchased from Changzhou Barege Biomedical Co.

Eight-week-old sexually mature female SPF-grade Sprague–Dawley rats weighing 220–250 g were obtained from the Shanghai Experimental Animal Centre. All animal experiments complied with the guidelines for the care and use of experimental animals at Changzhou University and were approved by the Animal Ethics Committee of Changzhou University (No. 20230310008).

### Preparation of HP407/HP188 temperature-sensitive hydrogel

Different proportions of HP407 and HP188 were added to sterile saline and stirred at 4°C for 24 h with a magnetic stirrer until the solution was clear. In order to analyze the range of gel-forming temperatures of HP407/HP188 polymers in different ratios, the samples were transferred to glass vials and the sol–gel transformation process was evaluated using the test tube inversion method. The temperature of the water bath was set from 12 to 42°C. Glass vials were first equilibrated in a 12°C water bath for 5 min and then heated at a rate of 1°C every 5 min. The gel temperature was determined with a thermometer gun or thermometer after the test tube was inverted for 1 min. To construct substrates for *in situ* gels at human physiological temperatures, the gel temperature should be maintained within the range of 30–36°C.

### Preparation of rCol III@HP407/HP188 temperature-sensitive hydrogel

Different ratios of rCol III were weighed and dissolved in a certain amount of sterile saline to configure a collagen solution (Since rCol III is acidic, the pH needs to be adjusted using 0.1 mol/l NaOH to make it neutral (pH = 7.0) during the use process for subsequent experiments), added to a certain amount of HP407/HP188 hydrogel, and stirred thoroughly at room temperature for 1 h. 0.01, 0.5, 1 and 2 wt% concentrations of the rCol III@HP407/HP188 hydrogels were prepared, respectively, in anticipation of subsequent experimental endeavors.

### Rheological evaluation

Rheological measurements were made using a rheometer (DHR 2, TA, USA), where the strain was applied to the sample through a fixture and the upper part of the sample was connected to a torque transducer through a fixture to observe the data of the sample’s response to the applied load. The rheological parameters such as dynamic viscosity, energy storage modulus G′, loss modulus G″ are calculated by measuring the rheological data such as shear rate, shear stress, oscillation frequency, stress–strain amplitude. In order to evaluate the rheological properties of temperature-responsive hydrogels, the kinetic viscosity of the samples was measured using a constant shear rate (0.4 s^−1^) at fixed temperatures of 25 and 37°C, respectively. The storage modulus G′ and loss modulus G″ of the hydrogels were measured at a constant frequency (6 Hz) as the temperature was increased from 12 to 42°C at a rate of 5°C/min.

### Microscopic morphology of temperature-sensitive hydrogels

The microscopic morphology of recombinant type III collagen, blank hydrogels and hydrogels with different concentrations of carrier proteins was evaluated using scanning electron microscopy (SEM) (TM3030Plus, Japan). The samples were firstly frozen at −80°C for 2 h and then dehydrated and dried using a lyophilizer. The surface of the freeze-dried samples was sprayed with gold and then scanned for subsequent analysis.

### *In vivo* release studies of rCol III

To begin with, two centrifuge tubes were taken and 1 ml of 2 wt% rColIII aqueous gel was added to one of them and 1 ml of 2 wt% rColIII aqueous solution was added to the other, with three parallel samples in each group. This tube is then placed into a thermostatic oscillator and spun at 120 rpm at 37°C. After observing the transformation of the sol into a gel, 5 ml of isothermal phosphate buffer solution (PBS) is added. Subsequently, 4 ml of release solution is aspirated and replaced with equal amounts of PBS at different time points. The protein concentration in the sample at each time point is determined using the aspirated release solution. The cumulative release rate of rCol III can then be calculated.

### Hemolysis test

In order to evaluate the compatibility of the hydrogel with blood, we determined the hemolysis rate. Here is the procedure: collect 500 μl of defibrillated rabbit blood, mix it with approximately 10 times the volume of PBS, and shake vigorously. Next, centrifuge the mixture for 15 min at a speed ranging from 1000 to 1500 rpm. Remove the supernatant and wash the precipitated erythrocytes with PBS solution two to three times, repeating the process until the supernatant is no longer red. The resulting erythrocytes were made into a 4% suspension with PBS solution and set aside. Positive and negative controls were incubated with deionized water and PBS, respectively. Four percent of erythrocyte suspension was introduced into each of the 500-μl samples, introducing the blank hydrogel and hydrogels with varying concentrations of carrier protein. The samples of erythrocyte suspension prepared above were incubated at 37°C for 1 h and then centrifuged at 2000 rpm for 15 min. Finally, the absorbance of each supernatant was measured at 540 nm. The hemolysis rate was calculated using the following formula:


$$\text{Hemolysis}\;\text{rate}\;(\%)=\left({\text{OD}}_{t}-{\text{OD}}_{n}\right)/\left({\text{OD}}_{p}-{\text{OD}}_{n}\right)\times 100$$

Here, ODt, ODn and ODp signify the absorbance values corresponding to the experimental (hydrogel group), negative control and positive control groups, respectively. In addition, the erythrocytes treated with each group were observed for any morphological changes.

### Cytotoxicity test

In order to assess the effect of temperature-responsive hydrogels on cell growth and survival, MTT (Thiazolyl blue) colorimetric method was used to evaluate the survival of hydrogels in samples with different concentration gradients. Hydrogel samples were extracted by incubating them in isothermal DMEM basal medium at 0.2 g/mL and maintained at 37°C for 24, 48 and 72 h. Logarithmically grown hESCs were inoculated in 96-well plates at a concentration of 5000 cells/well. After 24 h of incubation, the medium of hESCs was replaced with the extracted hydrogel infusion. Complete medium was used as negative control and complete medium containing 10% DMSO as positive control. They were then incubated for 24 h in a 5% CO_2_ incubator at 37 ± 1°C. Subsequently, the waste solution was removed, and after rinsing 2–3 times with PBS, 20 μl of 0.5 mg/ml MTT was added to each well and incubated for 4 h at 37°C in an incubator. MTT solution was carefully removed, and 150 μl of DMSO was subsequently added to each well. The plate was gently shaken to facilitate the dissolution of the formazan crystals. Analyses were carried out using a UV spectrophotometer at a wavelength setting of 490 nm. Cell viability was calculated using the following formula:


Cell viability (%)=OD490(sample)/OD490(control)×100

### Cell migration test

The cell scratch method was used to evaluate cell migration rates in samples with different concentration gradients. Cell migration motility, repair capacity and cell–cell interactions were investigated by scratching on cell monolayers and capturing images periodically through time-lapse microscopy. First mark the back of the 6-well plate with a marker pen, compared with a straightedge, and mark each hole in three equal horizontal or vertical thirds. Seed cells were seeded at approximately (5–15) × 10^5^ per well. The goal was to achieve 95–100% confluence after 24 h of culture. Three replicate wells per group. Draw three horizontal lines at the bottom of the well plate using a marker pen and a straightedge. After 24 h of cell culture, align the tip of a 10-μl pipette gun vertically with the well plate using a straighedge, and gently press down along the drawn horizontal line to form a scribe. Rinse the cells with PBS three times to remove the scribed cells, respectively, in both the experimental and control groups. Add 2 ml of serum-free medium prepared for the test and the control material to the experiment and control groups, while the blank control group only receives serum-free medium. The cells were cultured at 37°C, 5% CO_2_ incubator and photographed under a 20× microscope after 0, 6, 24 and 48 h with the intersection point of the transverse scratch line as the core. A software called ‘Image J’ was used to evaluate the size of the scratched area. Once the size of the scratched area is determined by this method, the cell migration rate for each group can be calculated.

### Vascularization test

HUVEC cells were selected through conventional cell passaging methods, utilizing cells from the third to fifth generations in good condition, and achieved 80% confluent within 24 h. HUVEC cells were then starved, replaced complete medium with an ECM medium containing 10% FBS and incubated for 24 h in an incubator. Thawing of low growth factor Matrigel matrix gel, remove the matrix gel from −20°C, thaw in 4°C and use a pre-cooled pipette or gun tip to mix the matrix gel to a homogeneous state. 50 μl of Matrigel containing reduced growth factors was added to a pre-frozen 96-well plate. The 96-well plate was transferred to a cell culture incubator and incubated at 37°C for 30 min to allow the basement membrane to form a gel. HUVEC cells that reached 80% confluence were digested, centrifuged and counted. Different concentrations of the test material were then added to the centrifuge tubes for resuspension. Using a Matrigel-coated 96-well plate containing reduced growth factors, 100 μl of cell suspension containing the sample to be tested was added to each well of the 96-well plate and inoculated with 1 × 10^4^ cells per well, with each sample comprising three parallel wells. The negative control was ECM complete medium and the positive control was ECM complete medium with bFGF concentration of 3 ng/ml. The petri dishes were incubated in an incubator and photographed for observation at 24 h time points.

### Western blot

hESCs were resuspended with complete medium containing 1 wt% rCol III and seeded at approximately (5–15) × 10^5^ cells per well using 6-well plates. The goal was to achieve 95–100% confluence after 24 h of incubation. After 24 h, the waste solution was removed and washed twice with pre-cooled PBS. PBS was aspirated, and Radio Immunoprecipitation Assay (RIPA) lysate (containing protease and phosphatase inhibitors) was added (0.1 ml/10^6^cells). Scrape the cells with a cell scraper, gently transfer the cells and lysate to a pre-cooled centrifuge tube. Centrifuge at 12 000 rpm for 20 min at 4°C, then gently aspirate the supernatant and transfer to a fresh, pre-cooled centrifuge tube on ice, which now contains the protein sample. Discard the precipitate, and the protein concentration was quantified using a BCA assay kit. Equal amounts of proteins were electrophoresed on a 12% Sodium Dodecyl Sulfate Polyacrylamide Gel Electrophoresis (SDS-PAGE) gel and then transferred to a polyvinylidene fluoride (PVDF) membrane. After blocking with 5% skimmed milk powder for 4 h at room temperature, the membranes were washed three times with Tris-Buffered Saline-Tween (TBST). After overnight incubation with TGF-β1 antibody at 4°C, the membranes were washed three times with TBST. Next, the membrane was treated with horseradish peroxidase-labeled goat anti-rabbit IgG for 1 hour at room temperature. This was followed by three thorough washes with TBST. The corresponding proteins on the membrane were visualized using enhanced chemiluminescence (ECL) chemiluminescent reagents. Anti-β-actin antibody was used as a protein loading control. Quantitative analyses were performed using a Tanon 5200 Multi (Tanon, China) and shown as relative expression of β-actin.

### Construction of rat IUA model

In this study, 8-week-old sexually mature female SPF-grade Sprague–Dawley rats weighing 220–250 g were obtained from the Shanghai Experimental Animal Center. One week of rat acclimatization culture was conducted. To ensure that the animals were as close as possible to the same hormone level during the curettage operation, the rats were tested for estrus using the vaginal decidual cell method prior to the curettage procedure. Typically, the metestrus phase is chosen for the surgical operation of scraping when the cell morphology under the inverted microscope consists of anucleate keratinized epithelial cells, and the levels of estrogen and progesterone in rats at this stage are higher, making the uterine state relatively full, which makes it more suitable for the scraping operation. Under 4% isoflurane induction anesthesia and 2% isoflurane surgical maintenance anesthesia, the skin of the abdominal wall of the rats was incised longitudinally for about 3 cm at a point 2–3 cm above the pubic symphysis, and then the tissues were incised one by one to enter the abdominal cavity, and the Y-shaped uterus of the rats was slowly withdrawn. A 2-mm transverse incision was made 0.5 cm from the upper end of the cervical opening. The lining of the uterus is scraped with a medicated spoon until the walls of the uterine cavity become rough and congested, and then the uterine incision is sutured. After cleaning the abdominal cavity with saline, the abdominal muscles and skin were closed in layers with sutures. The sham-operated group was sutured as soon as the uterine incision was made.

### Evaluation of hydrogel retention time in the body

To evaluate the retention time of HP407/HP188 temperature-responsive hydrogel *in vivo*, 100 μl of Cy7 saline and 100 μl of Cy7 hydrogel (Cy7 concentration of 1 mg/ml) were unilaterally injected into the uterine cavity of IUA-modeled rats. Upon administration, for the initial 10 days, the rat uterus was observed and the uterine surface was rinsed with saline. Finally, fluorescent images of the intact uterus were acquired using a small animal *in vivo* optical imaging system (Tanon ABL X5).

### Evaluation of the safety of hydrogels *in vivo*

After screening the rats in the actinic phase, they were divided into different groups using a randomization method. These groups included: (i) control group, no scraping; (ii) for the hydrogel group, 100 μl of hydrogel was injected into the rat uterus after IUA modeling. Blood was collected from rats on day 8 to evaluate blood safety.

### Hydrogel implantation in rats

After screening the rats in the actinic phase, they were divided into eight groups using a randomization method. These groups include: (i) IUA group (Group 1), consisting of rats not treated with IUA; (ii) Sham operation group (Group 2), uterine incision only, no curettage; (iii) CLHA group (Group 3), an IUA model was established and both uteri were injected with 100 μl of cross-linked sodium hyaluronate gel; (iv) blank hydrogel group (Group 4), the IUA model was established and both uteri were injected with 100 µl of blank hydrogel; (v) for the 0.01 wt% Col-gel group (Group 5), the IUA model was established and 100 μl of 0.01 wt% hydrogel was injected into both uteri; (vi) for the 0.5 wt% Col -gel group (Group 6), the IUA model was established and 100 μl of 0.5 wt% hydrogel was injected into both uteri; (vii) for the 1 wt% Col-gel group (Group 7), the IUA model was established and 100 μl of 1 wt% hydrogel was injected into both uteri; (viii) for the 2 wt% Col-gel group (Group 8), the IUA model was established and 100 μl of 2 wt% hydrogel was injected into both uteri. The rats’ estrous cycles were monitored daily throughout the experiment. At the end of the two estrous cycles, the rats were executed and the uterus was removed.

### Assessment of endometrial gland number and endometrial thickness

Following the removal of the uterus, it was immediately immersed in 4% paraformaldehyde tissue fixative. Afterward, the tissue was carefully processed through a series of steps, ultimately resulting in paraffin sectioning, which was then used for hematoxylin-eosin (HE) staining. A procedure using the NPD.view2 image viewing software was then employed to analyze the pathology images, which provided invaluable insights into the number of glandular tissue present and the endometrial thickness.

### Assessment of anti-fibrotic effect

The uterus was excised and immersed in 4% paraformaldehyde tissue fixative. The tissue was then prepared for paraffin embedding and sections were cut for staining with the Masson and Sirius red staining technique. Following this, images of the tissue sections were acquired using NPD.view2 image viewing software with four randomly selected fields of view. Image analysis using the NPD.view2 software was performed, with a specific focus on the presence of fibrosis and the area of type I collagen in polarized light, and the resulting data were assessed using Image J software.

### Evaluation of the effectiveness of immunohistochemistry

The uterus was collected and preserved using the 4% paraformaldehyde tissue fixative. Then, TGF-β1, CD31, IL-6 and TNF-α antigen–antibody reactions were carried out by paraffin sectioning technique. The acquired pathological images were examined through NPD.view2 image viewing software, with four randomly selected fields of view. Lastly, fibrosis and vascularization were inflammatory factors evaluated using Image J software.

### Statistical analyses

The mean ± standard deviation is used to express all experimental data. A one-way analysis of variance was utilized to measure the statistical significance for multiple comparisons. ImageJ was used to perform quantitative analyses of all stained images, and analysis of the data was conducted with Origin 2022 or GraphPad Prism 8. *P* < 0.05 was deemed statistically significant.

## Results and discussion

### Preparation and characterization of temperature-responsive hydrogels

**Figure 2.**
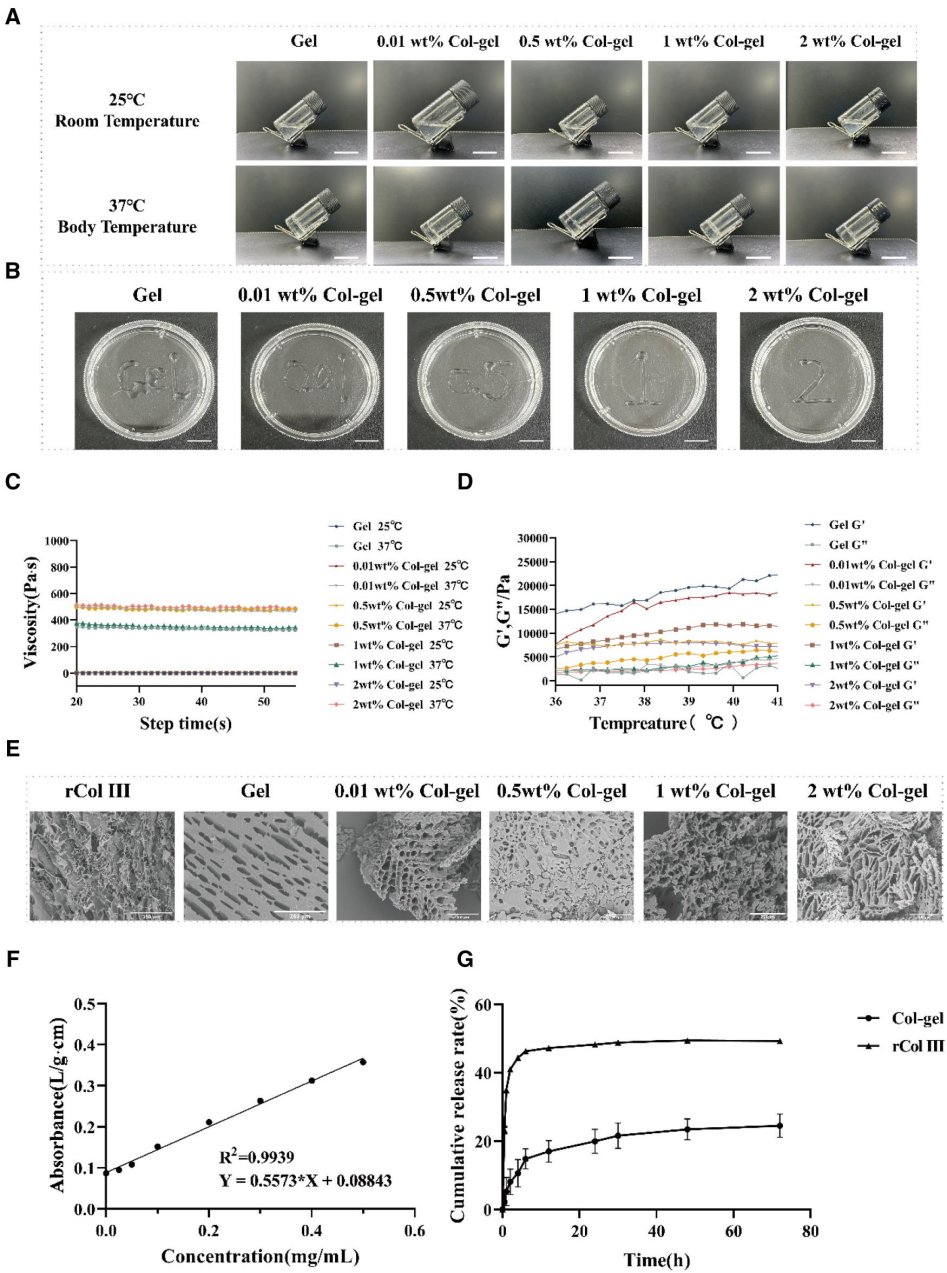
Preparation and characterization of temperature-responsive hydrogels. (**A**) Different morphologies of hydrogels obtained at 25 and 37°C environments. Scale bar: 2 cm. (**B**) Injectability of hydrogels with different collagen concentrations at room temperature (25°C). Scale bar: 2 cm. (**C**) Kinetic viscosity of hydrogels at 25 and 37°C. (**D**) The storage modulus G′ of the hydrogel is greater than the loss modulus G″ at a constant frequency (6 Hz) when the temperature is 36–42°C, indicating that the hydrogel solidifies at this temperature. (**E**) Scanning electron microscope (SEM) images of rCol III, gel and Col-gel containing different collagen concentrations. Scale bar: 250 μm. (**F**) UV-Vis calibration curves for known amounts of rCol III with corresponding linear fits. (**G**) Cumulative release of rCol III from Col-gel.

Hydrogels that respond to temperature were created using varying proportions of HP407 and HP188. To find the ideal gel temperature, several ratios were evaluated, as detailed in Table S1. The experimental findings indicated that the optimal hydrogel formulation comprised 20 wt% HP407 and 7.5 wt% HP188. To evaluate the solubility of rCol III, the compound was incorporated into the hydrogel system. Ultimately, it was determined that four hydrogel formulations were used, all of which exhibited good temperature responsiveness and injectability (Figure 2A and B and Figure S1). The percentage of rCol III was 0.01 wt% Col-gel, 0.5 wt% Col-gel, 1 wt% Col-gel and 2 wt% Col-gel for subsequent experiments.

The rheological analyses of hydrogels demonstrated that the kinetic viscosity of hydrogels at human physiological temperature (37°C) was consistently significantly higher than that at ambient state (Figure 2C). Under the same conditions, the kinetic viscosity increased with the increasing content of rCol III, suggesting that collagen has the effect of enhancing the kinetic viscosity of the hydrogel, which contributes to the prolonged retention time of the hydrogel *in vivo*. At a constant frequency (6 Hz), when the temperature ranged from 36 to 42°C, the energy storage modulus G′ was consistently greater than the loss modulus G″, indicating the formation of a relatively stable 3D mesh structure with elastic and solid-like rheological properties (Figure 2D and Figure S2).

The freeze-dried fibers of recombinant type III collagen, blank hydrogels and different concentrations of recombinant type III collagen hydrogels were frozen at −80°C for 2 h and then dehydrated and dried using a freeze-dryer. Finally, photographic analysis by SEM showed that the recombinant type III collagen behaved as fibrils and the hydrogel had a porous structure (Figure 2E), with no significant difference between the pore size and the collagen content.

The aqueous solution of pure recombinant type III collagen is highly mobile, making it difficult to maintain *in situ* for long periods of time. The cumulative release of collagen alone shows a rapid increase in the initial period (0–6 h), with a release rate close to 50%, and then enters a slow release phase, eventually reaching a release plateau at around 24 h, with a cumulative release of about 40–50%. However, when compounded with the hydrogel, the cumulative release of collagen showed a rapidly rising trend at the initial stage (0–6 h), with a release rate close to 15%, and then entered a slow release stage, eventually reaching a release plateau period at about 72 h, with a cumulative release of about 20%. Thus, the protein-carrying hydrogel promotes the sustained release of collagen at the lesion site, thereby improving the efficacy of the treatment (Figure 2F and G).

### Security evaluation

**Figure 3.**
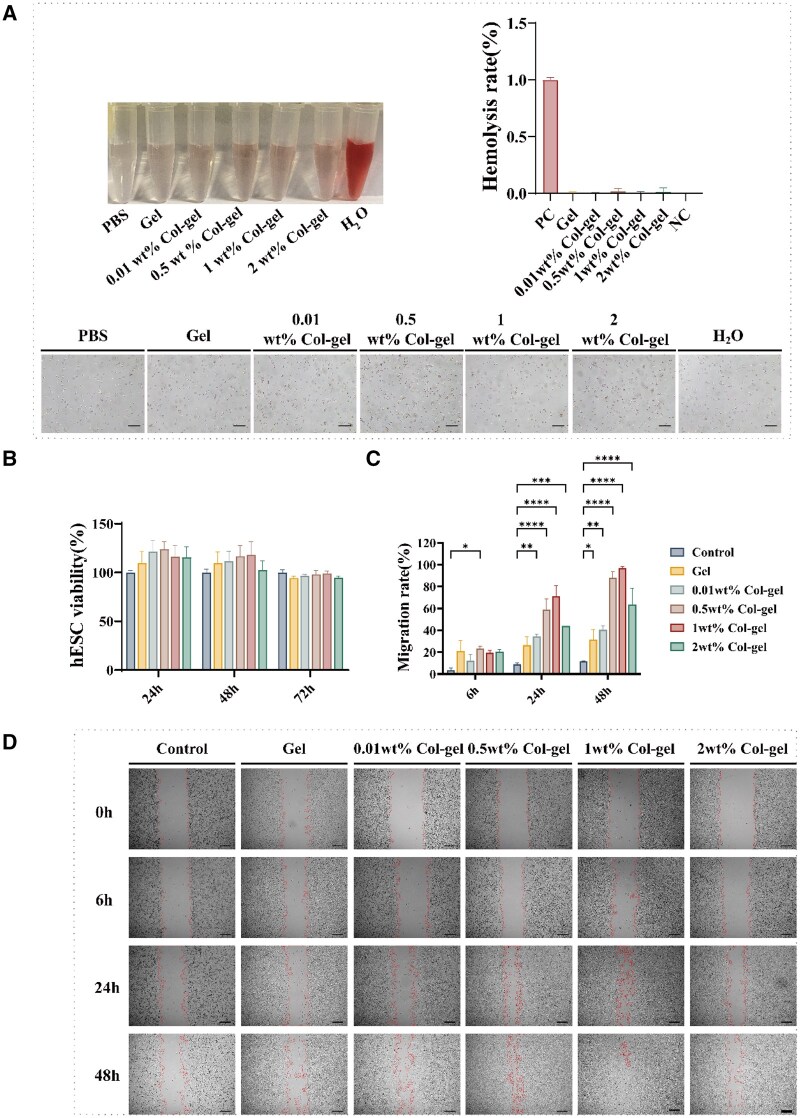
Biocompatibility assessment of temperature-responsive hydrogels. (**A**) Hemolysis test was used to assess the hemocompatibility of the hydrogels. Deionized water was used as a positive control and PBS as a negative control. Scale bar: 1000 μm. (**B**) The effect of hydrogel on cell viability of hESCs was assessed by the MTT method. Control cells were not exposed to any material extraction medium. (**C** and **D**) The mobility of hESCs in samples with different concentration gradients was evaluated by the cell scratch method. Scale bar: 100 μm.

Biocompatibility testing assesses the absence of adverse effects when biomaterials come into contact with the human body, thereby confirming the product’s safety for patients [41]. Given that curettage results in direct contact between the hydrogel and the injury site, it is essential to evaluate the hydrogel’s safety. Therefore, we performed a hemolysis test, a blood safety test and a cell viability test. The findings, depicted in (Figure 3A), indicate the state of red blood cells following treatment with Gel and different concentrations of Col-gel was nearly identical to that observed after PBS treatment. Moreover, the hemolysis rate of Gel and Col-gel was extremely lower than that of PBS, signifying excellent blood compatibility of the samples. As shown in Figure S8, there were no significant changes in routine blood and blood biochemistry tests after the eighth day of Col-gel implantation in rats compared with normal rats. The main liver function indicators, albumin and alkaline phosphatase, etc., and the main renal function indicators, urea/urea nitrogen and creatinine, etc., did not show significant changes. This shows that hydrogels do not have any significant acute toxicity. In addition, when the hydrogel extracts were co-cultured with hESCs, there was no significant difference in cell viability compared to the control (Figure 3B), and there was also a promotion of cell growth. These *in vitro* safety studies suggest that hydrogel can be used as a safe and non-toxic *in vivo* implant material.

### *In vitro* biological functions of col@HP407/HP188 hydrogels

**Figure 4.**
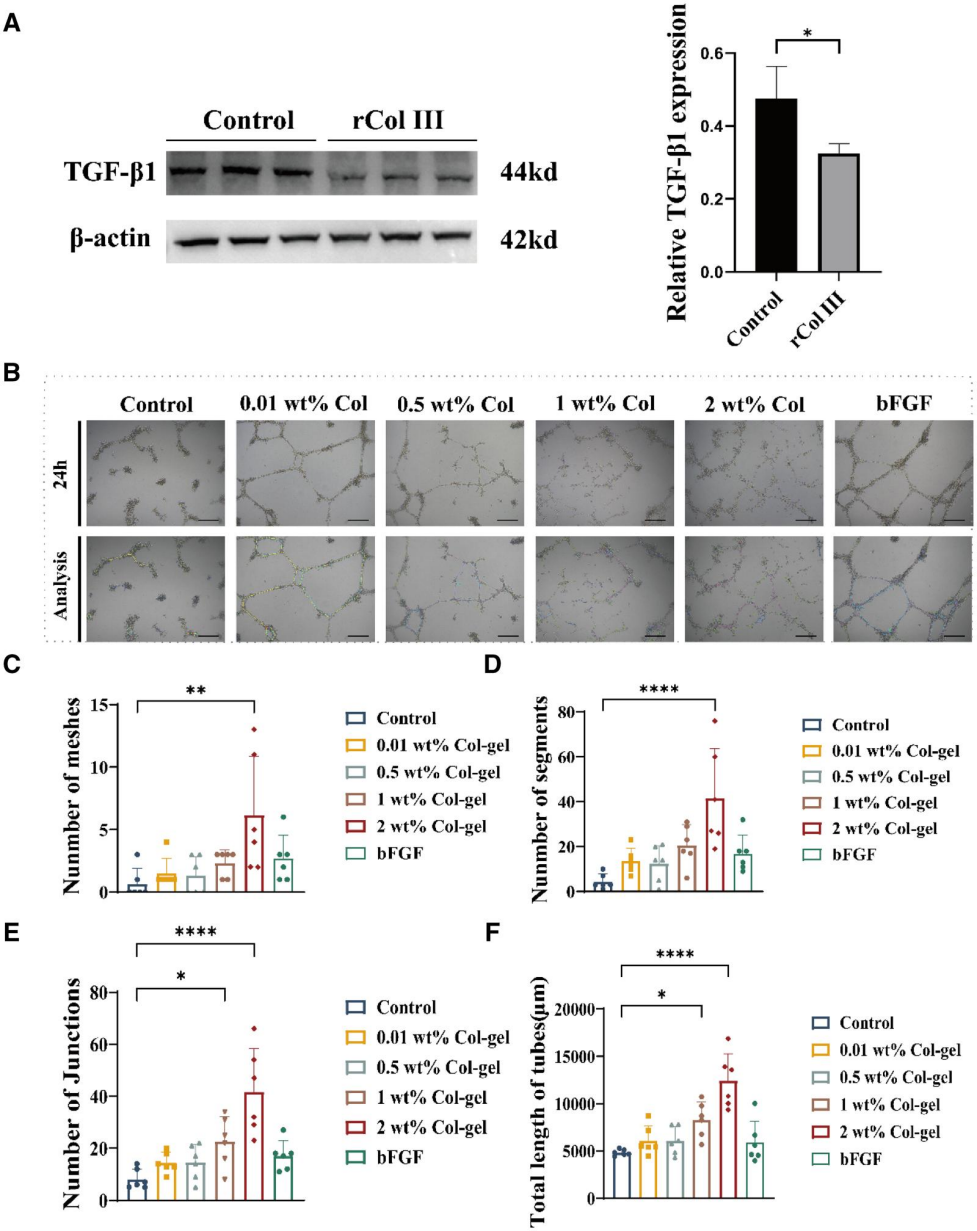
*In vitro* bioactivity assessment of rCol III temperature-responsive hydrogels. (**A**) Western blot analysis of hESCs treated with rCol III for 24 h (blots were cropped). (**B**) Angiogenesis was measured with the angiogenesis test. Micrographs were analyzed with angiogenesis analyzer for image J. Scale bar: 100 μm. (**C**–**F**) Quantitative analysis of capillary formation parameters, including the number of junctions, number of segments, number of meshes and total capillary length, was performed with an angiogenesis analyzer (angiogenesis analyzer for image J).

Cell migration plays a key role in tissue repair and regeneration. When tissue is damaged or irritated, cell migration can help new cells to fill in the damaged areas [42, 43]. The results showed (Figure 3C and D) that when different concentrations of hydrogels were co-cultured with hESCs cells after immersion, they had a significant effect of promoting cell migration at 24/48 h, in which we can see an interesting phenomenon that the blank hydrogel group had a certain effect of promoting cell migration in different time periods, which may be attributed to the fact that Poroxam188 showed potential to protect the cell membrane integrity and promote cell recovery in the cell damage model showed the potential to protect cell membrane integrity and promote cell recovery [44, 45]. rCol III was found to modulate the expression of key TGF-β1 associated with fibrotic disease [46–48]. The expression of TGF-β1 in hESCs treated with recombinant type III collagen was lower than that in the control group (Figure 4A), suggesting that recombinant type III collagen has anti-endometrial fibrosis properties. rCol III also has the potential to promote wound angiogenesis [49]. To this end, we performed an *in vitro* angiogenesis experiment. Initially, we investigated the effects of different concentrations of rCol III on the angiogenesis of HUVECs by co-culturing HUVECs with different concentrations of rCol III for 24 h (Figure 4B). The results showed that rCol III exhibited a significant trend in total tube length, number of reticular pores, number of junctions and number of segments in this cell line compared to the control group (Figure 4C–F), which suggests that rCol III has a promotional effect on blood vessel formation.

### Therapeutic effect of col@HP407/HP188 hydrogel on IUA rats *in vivo*

**Figure 5.**
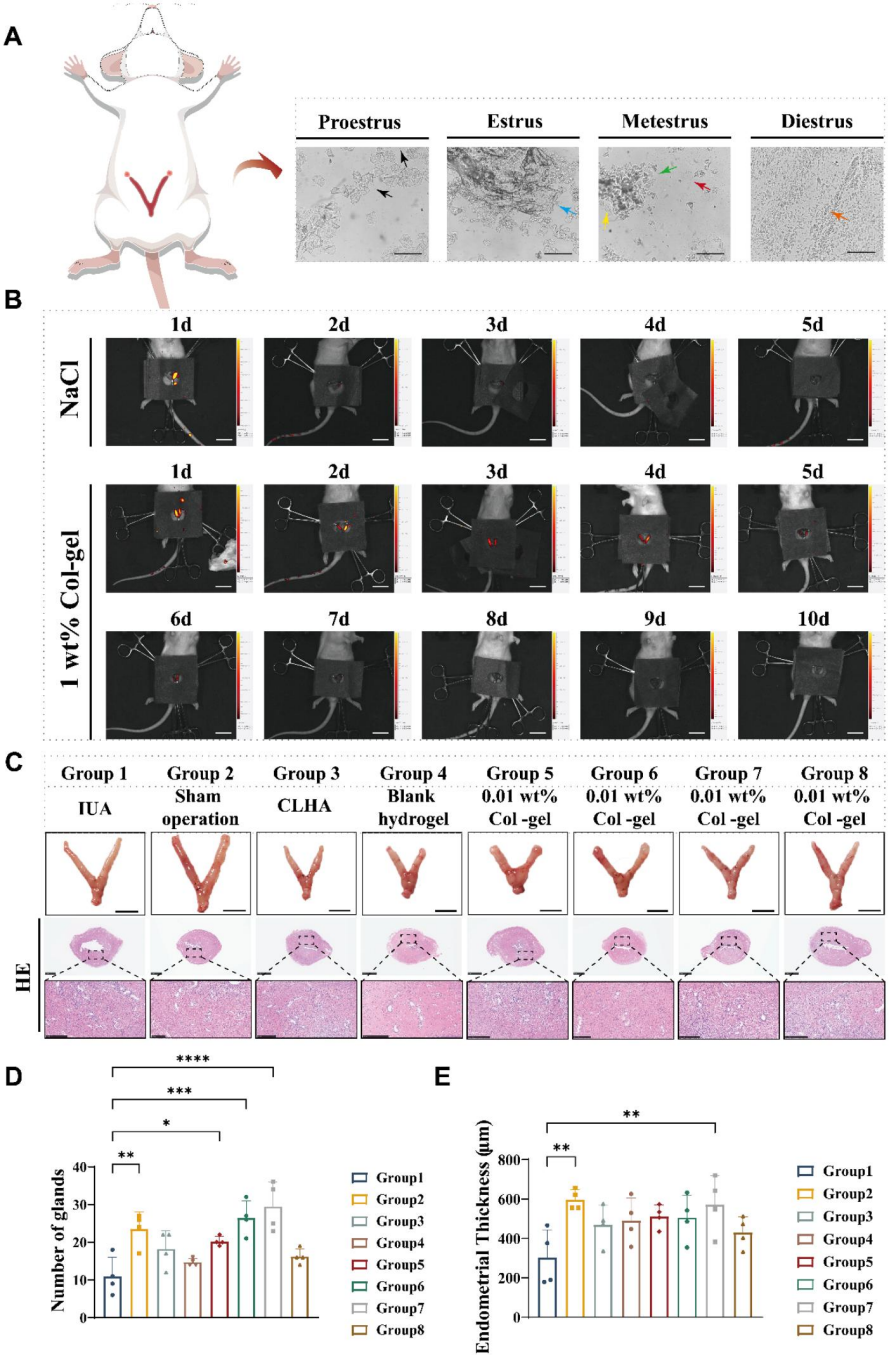
Therapeutic effects of rCol III temperature-responsive hydrogels in IUA rats. (**A**) Screening for rat motility. Scale bar: 1000 μm. (**B**) Fluorescent images of retention time of hydrogels in rats. Scale bar: 3 cm. (**C**) Corresponding HE staining. Scale bar: 1 mm, 250 μm. (**D**) Number of endometrial glands. (**E**) Quantitative analysis of endometrial thickness.

*In vivo* evaluation of the therapeutic efficacy of hydrogels using the IUA rat model. Since hormones affect the endometrium [50–52], in order to validate the effectiveness in rats at the same hormone level, rats in the actinic phase were selected, when the uterine cavity was full and better able to accommodate the scraping spoon (Figure S7). We determined the rat estrous cycle by vaginal smear method [53], and the morphology of vaginal exfoliated cells varied in all four phases: preestrus nucleated epithelial cells, estrus-nucleated keratinized epithelial cells, late estrus small numbers of leukocytes and keratinized cells, and interestrus large numbers of leukocytes. Rats in estrus were randomly selected for surgery (Figure 5A and Figure S7). Cervical subluxation and uterine sampling were performed after two estrous cycles. Fluorescence imaging showed that the hydrogel retarded the retention of encapsulated rCol III *in vivo* (Figure 5B) and was able to cover the critical repair phase of the endometrium [54, 55].

HE staining showed significant endometrial thinning and adhesions in the IUA group. Hyaluronic acid gel (CLHA), blank gel, and hydrogel groups with different collagen concentrations (Col-gel) showed good anti-adhesion effects without causing significant histomorphological changes (Figure 5C and Figure S3). Statistical analysis showed that the number of glands in the 1 wt% Col-gel group was similar to that of the sham-operated group and significantly higher than that of the IUA group (*P* < 0.0001) (Figure 5D). Gland recovery was relatively better in the low concentration hydrogel group (0.01 wt% Col-gel, 0.5 wt% Col-gel), with significant differences compared to the IUA group, and poorer in the CLHA group relative to the IUA group. Endometrial thickness was significantly higher in the 1 wt% Col-gel group than in the IUA group (*P* < 0.01), and the rest of the test groups had some restorative effect on endometrial thickness (Figure 5E). In conclusion, 1 wt% Col-gel was superior to CLHA in endometrial regeneration. 1 wt% Col-gel was comparable to the sham-operated group in terms of intrauterine test recovery.

**Figure 6.**
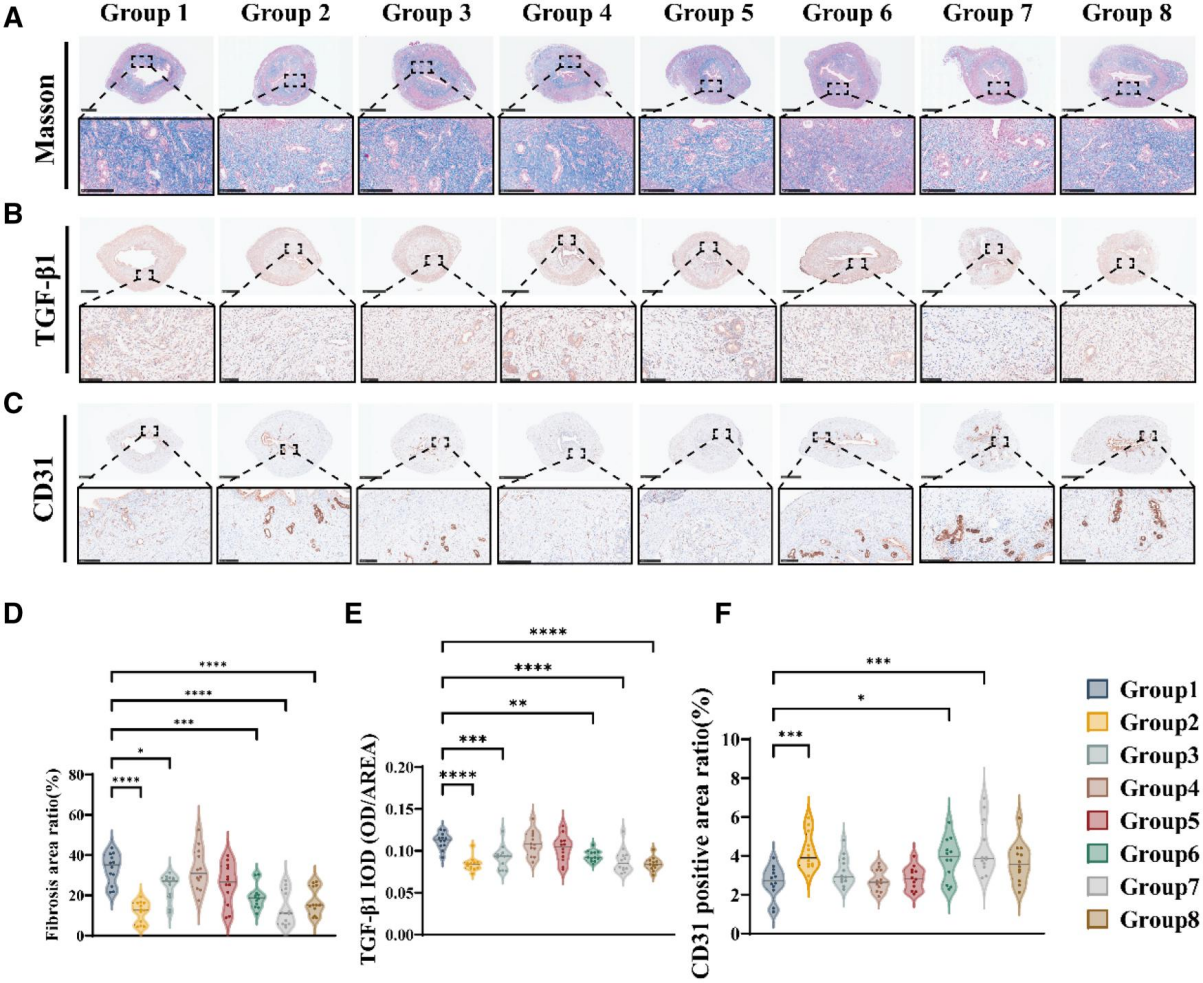
(**A**) Corresponds to Masson staining. Scale bar: 1 mm, 250 μm. (**B**) Corresponding TGF-β1 immunohistochemical staining. Scale bar: 1 mm, 100 μm. (**C**) Corresponding CD31 immunohistochemical staining. Scale bar: 1 mm, 250 μm. (**D**) Quantitative analysis of fibrosis. (**E**) Quantitative analysis of TGF-β1 protein expression levels. (**F**) Quantitative analysis of CD31 protein expression levels.

Inhibition of excessive collagen fiber deposition and restoration of endometrial blood supply are key factors in endometrial repair [56–58]. By looking at Masson stained collagen fibers (blue) it was shown that the 0.5 wt% Col-gel, 1 wt% Col-gel, 2 wt% Col-gel and CLHA groups had a significant antifibrotic effect (Figures 6A and D and Figure S4). TGF-β1 immunohistochemical staining showed that the mean positive expression of TGF-β1 was significantly lower in the 0.5 wt% Col-gel, 1 wt% Col-gel, 2 wt% Col-gel, and CLHA groups compared to the IUA group (Figure 6B and E and Figure S5), with a significant difference relative to the IUA group. CD31 immunohistochemical staining showed that the vascularized area was significantly higher in the 0.5 wt% Col-gel group and the 1 wt% Col-gel group than in the IUA group (*P* < 0.05; *P* < 0.001), and was comparable to that of the sham-operated group (Figure 6C and F and Figure S6). This phenomenon could be elucidated by a synthesis of the literature, which revealed that high molecular weight hyaluronic acid may impede angiogenesis [59], whereas HP188 has a protective effect on damaged microvascular endothelial cells [60, 61]. In conclusion, 1 wt% Col-gel was more effective than CLHA and the rest of the donor group in intrauterine test recovery.

**Figure 7.**
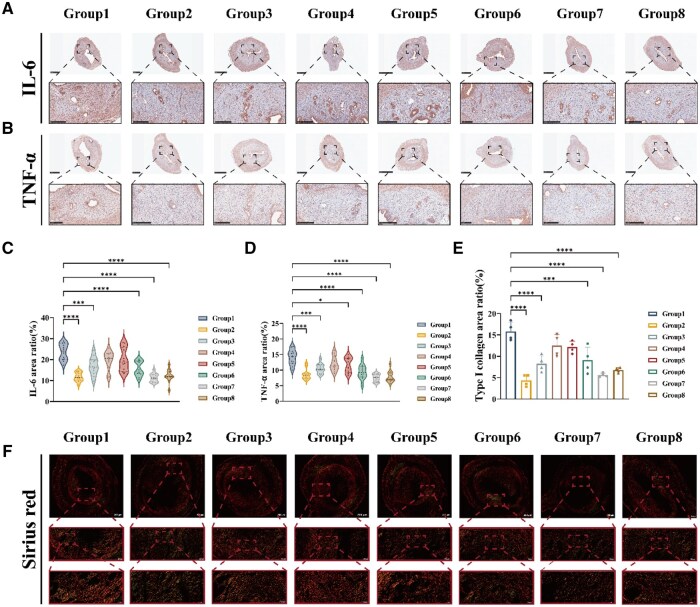
(**A**) Corresponds to IL-6 staining. Scale bar: 1 mm, 250 μm. (**B**) Corresponding TNF-α immunohistochemical staining. Scale bar: 1 mm, 250 μm. (**C**) Quantitative analysis of IL-6 protein expression levels. (**D**) Quantitative analysis of TNF-α protein expression levels. (**E**) Type I collagen area ratio. (**F**) Corresponds to Sirius red staining. Scale bar: 250, 100, 50 μm.

Inflammatory factors IL-6 and TNF-α play key regulatory roles at the end stage of endometrial repair [62–64]. At the end of repair, the expression of IL-6 and TNF-α needs to be moderately reduced to promote the subsidence of inflammation and the restoration of tissue homeostasis. The reduction of IL-6 helps to avoid tissue damage and fibrosis caused by persistent inflammation. By observing the results of IL-6 immunohistochemical staining, it was evident that the 0.5 wt% Col-gel, 1 wt% Col-gel, 2 wt% Col-gel and CLHA groups had a significant inhibition of the inflammatory response relative to the IUA group. The mean positive expression of IL-6 was significantly lower in the 0.5 wt% Col-gel, 1 wt% Col-gel, 2 wt% Col-gel and CLHA groups compared to the IUA group (Figure7A and C), which was significant relative to the IUA group. The decrease in the level of TNF-α, on the other hand, prevented the over-immune response and apoptosis and ensured that endometrial structures and functions were completely restored. Immunohistochemical staining of TNF-α showed that both the Col-gel and CLHA groups at different concentrations had a significant inhibitory effect on the inflammatory response relative to the IUA group (Figure7B and D). The dynamic regulation of both groups at the end of the repair stage is an important sign of the successful completion of the repair process.

Sirius red staining combined with polarized light microscopy shows type I collagen in orange/red and type III collagen in green. By analyzing the ratio of type I to type III collagen, scar formation can be assessed. The lower the proportion of type I collagen, the better the repair results [65, 66]. From the results, there was a significant downward trend in the percentage of type I collagen in the 0.5 wt% Col-gel, 1 wt% Col-gel, 2 wt% Col-gel, and CLHA groups compared to the IUA group (Figure 7E and F), suggesting a significant efficacy in combating recalcitrant connective tissue.

## Conclusion

This study investigated the application potential of rCol III in preventing IUA and aiding functional recovery of the endometrial using the HP407/HP188 temperature-responsive hydrogel as a scaffold, compounded with rCol III. The results showed that rCol III positively impacted adhesion prevention by stimulating the migration of hESCs to the injury site, facilitating angiogenesis, and reducing the expression of the fibrosis factor TGF-β1. *In vitro* trials have ultimately validated that the rCol III temperature-responsive hydrogel exhibits superior biological descriptive characteristics and remarkable maneuverability. Its unique ability to transform from a Sol–gel state into a semi-solid gel state at human body temperature enables it to sustain its structure within the uterine cavity. *In vivo* experiments demonstrated the effectiveness of rCol III temperature-responsive hydrogels. It was observed that collagen-free hydrogels, though capable of partially preventing uterine adhesions, showed limited effectiveness in restoring endometrial dysfunction. On the other hand, various concentrations of collagen hydrogel had different effects on endometrial recovery, with 1 wt% Col-gel emerging as the most effective based on experimental results. This hydrogel was effective in preventing adhesions, promoting endometrial function recovery, and significantly reducing abnormal collagen fiber deposition, thereby inhibiting scar formation. Consequently, we developed a safe and effective hydrogel for the prevention of IUA.

## Supplementary Material

rbaf013_Supplementary_Data
