# Supplementary material for: Development and functional evaluation of recombinant type III collagen intrauterine implant gel
Source: Regen Biomater. 2025 Mar 17;12:rbaf013. doi: 10.1093/rb/rbaf013 (PMC11975284; doi:10.1093/rb/rbaf013)
Supplement: rbaf013_Supplementary_Data [file rbaf013_supplementary_data.docx]

**Supporting Information**

**Development and Functional Evaluation of Recombinant Type III Collagen Intrauterine Implant Gel**

**Xinhui Wang^1,2^, Xiaoju Fan^2^, Yuanxin Zhai^2^, Jie Li^2^, Huilin Sun^2^, Jie Li^2^,Hao Le****^2^,Feng Zhang^2^, Li Zhang^1^，Jianhao Wang^1, *^, Yun Chu^2, *^, Pengfei Cui^1, *^**

**a. School of Pharmacy, Changzhou University, Changzhou 213164, P. R. China**

**b. Jiangsu Trautec Medical Technology Co., Ltd, Changzhou 213200, P. R. China**

***Correspondence:** **minuswan@cczu.edu.cn for Prof. Wang Jianhao; chuyun@trautec.com.cn for Dr. Chu Yun;** **cuizy1990@cczu.edu.cn for Prof. Cui Pengfei**

Table S1. The gelation temperature of different hydrogel formulations.

| **F127/F68**  **(wt%)** | **Gelling**  **Temperature**  **(℃）** | **F127/F68（wt%）** | **Gelling**  **temperature（℃）** | **F127/F68（wt%）** | **Gelling**  **temperature（℃）** | **F127/F68（wt%）** | **Gelling**  **temperature（℃）** |
| --- | --- | --- | --- | --- | --- | --- | --- |
| **20/0** | **23** | **21/0** | **22** | **22.5/0** | **20** | **25/0** | **19** |
| **20/4** | **24** | **21/4** | **23** | **22.5/4** | **20** | **25/4** | **19** |
| **20/5** | **27** | **21/5** | **25** | **22.5/5** | **22** | **25/5** | **20** |
| **20/5.5** | **29** | **21/5.5** | **28** | **22.5/5.5** | **25** | **25/5.5** | **21** |
| **20/6** | **32** | **21/6** | **29** | **22.5/6** | **27** | **25/6** | **21** |
| **20/7.5** | **33** | **21/7.5** | **30** | **22.5/7.5** | **28** | **25/7.5** | **22** |
| **20/10** | **36** | **21/10** | **32** | **22.5/10** | **29** | **25/10** | **22** |

| **F127/F68**  **(wt%)** | **Gelling**  **Temperature**  **(℃）** | **F127/F68（wt%）** | **Gelling**  **temperature（℃）** | **F127/F68（wt%）** | **Gelling**  **temperature（℃）** | **F127/F68（wt%）** | **Gelling**  **temperature（℃）** |
| --- | --- | --- | --- | --- | --- | --- | --- |
| **20/0** | **23** | **21/0** | **22** | **22.5/0** | **20** | **25/0** | **19** |
| **20/4** | **24** | **21/4** | **23** | **22.5/4** | **20** | **25/4** | **19** |
| **20/5** | **27** | **21/5** | **25** | **22.5/5** | **22** | **25/5** | **20** |
| **20/5.5** | **29** | **21/5.5** | **28** | **22.5/5.5** | **25** | **25/5.5** | **21** |
| **20/6** | **32** | **21/6** | **29** | **22.5/6** | **27** | **25/6** | **21** |
| **20/7.5** | **33** | **21/7.5** | **30** | **22.5/7.5** | **28** | **25/7.5** | **22** |
| **20/10** | **36** | **21/10** | **32** | **22.5/10** | **29** | **25/10** | **22** |


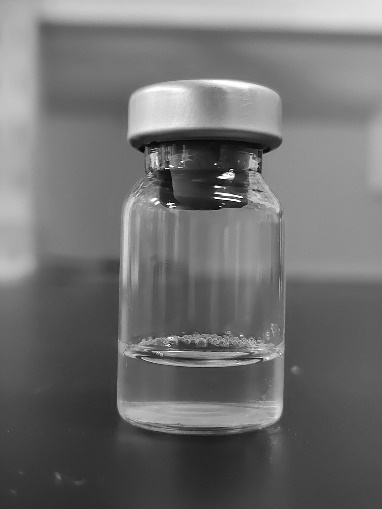

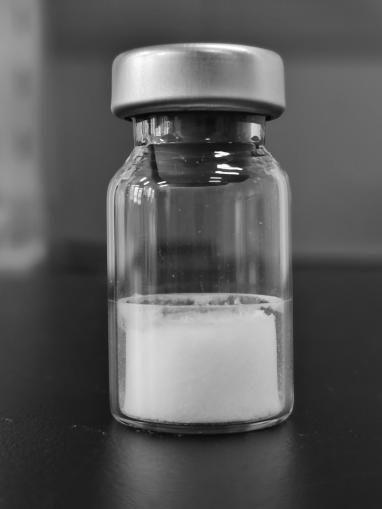


**H_2_O**

Figure S1. Image of recombinant type III collagen soluble in water.


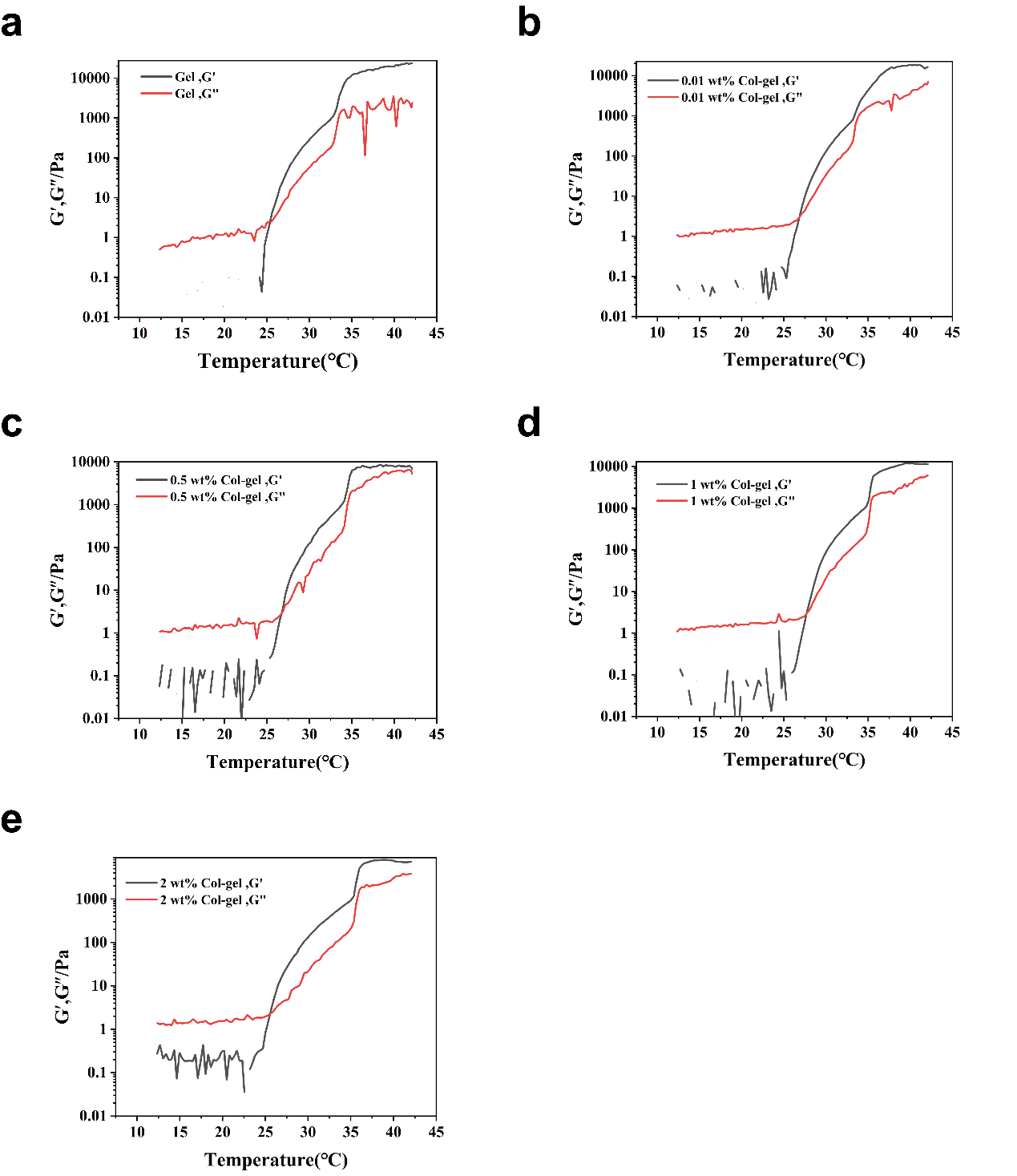


Figure S2. a-e) Effect of temperature on gel G′ and G″.


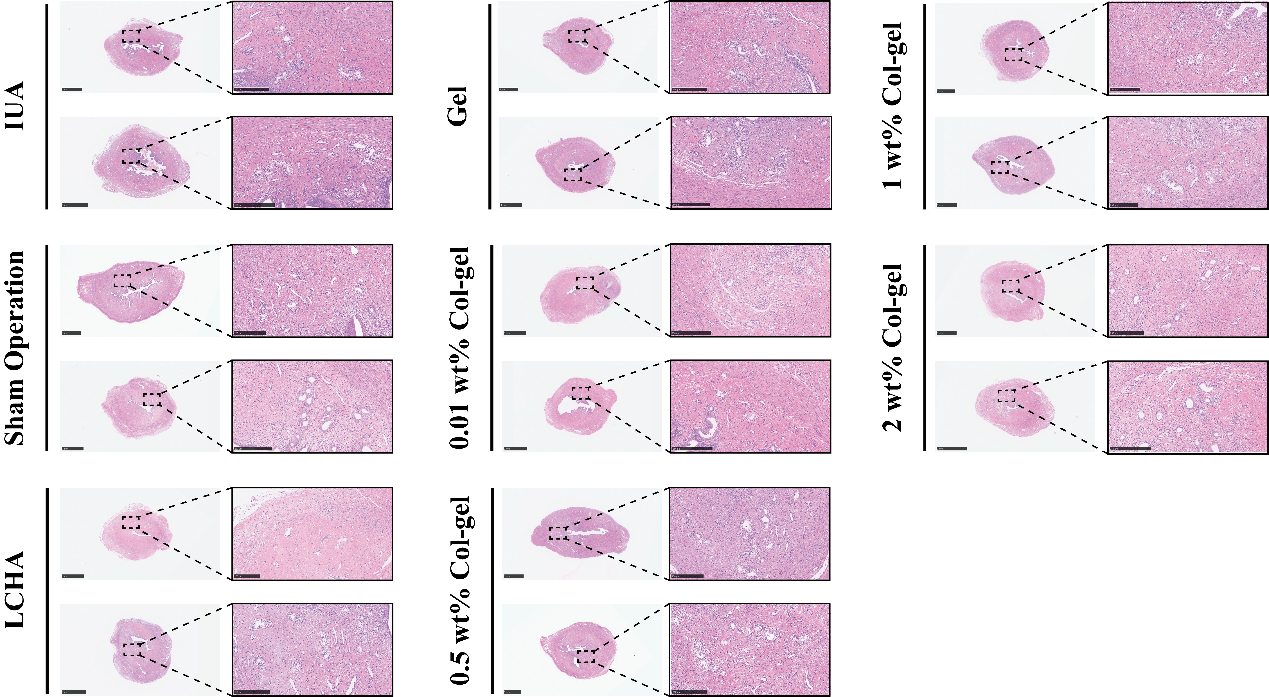


Figure S3. Corresponding HE stain. Scale bar: 1 mm, 250 µm.


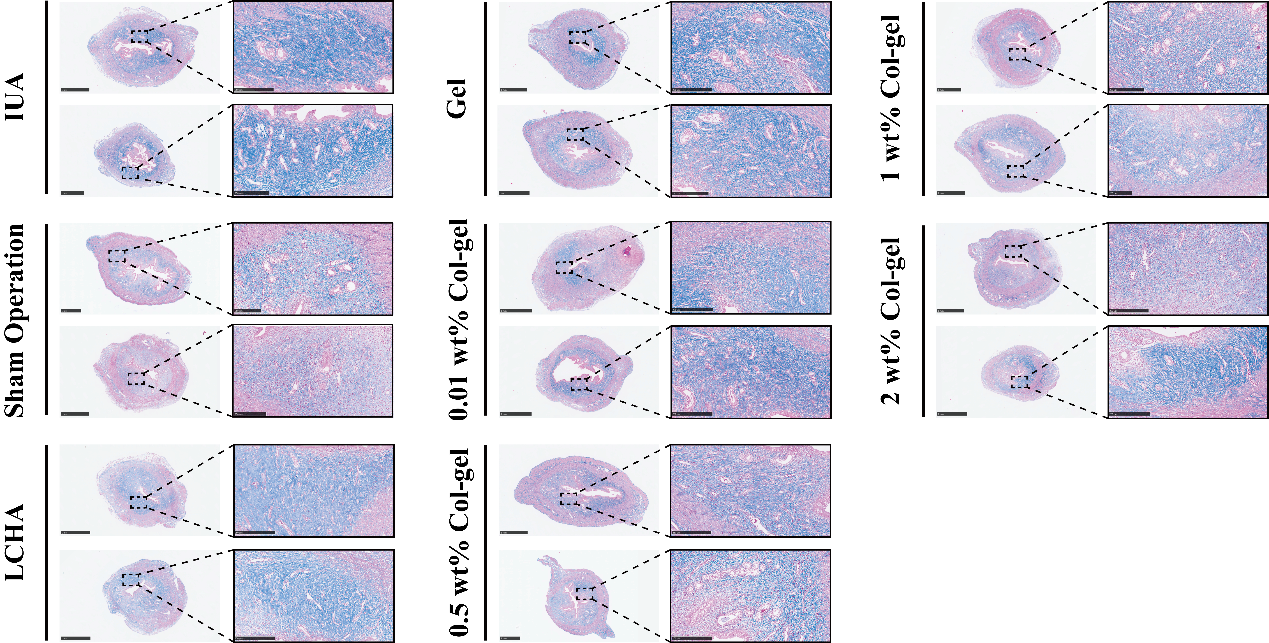


Figure S4. Corresponding Masson stain. Scale: 1 mm, 250 µm.


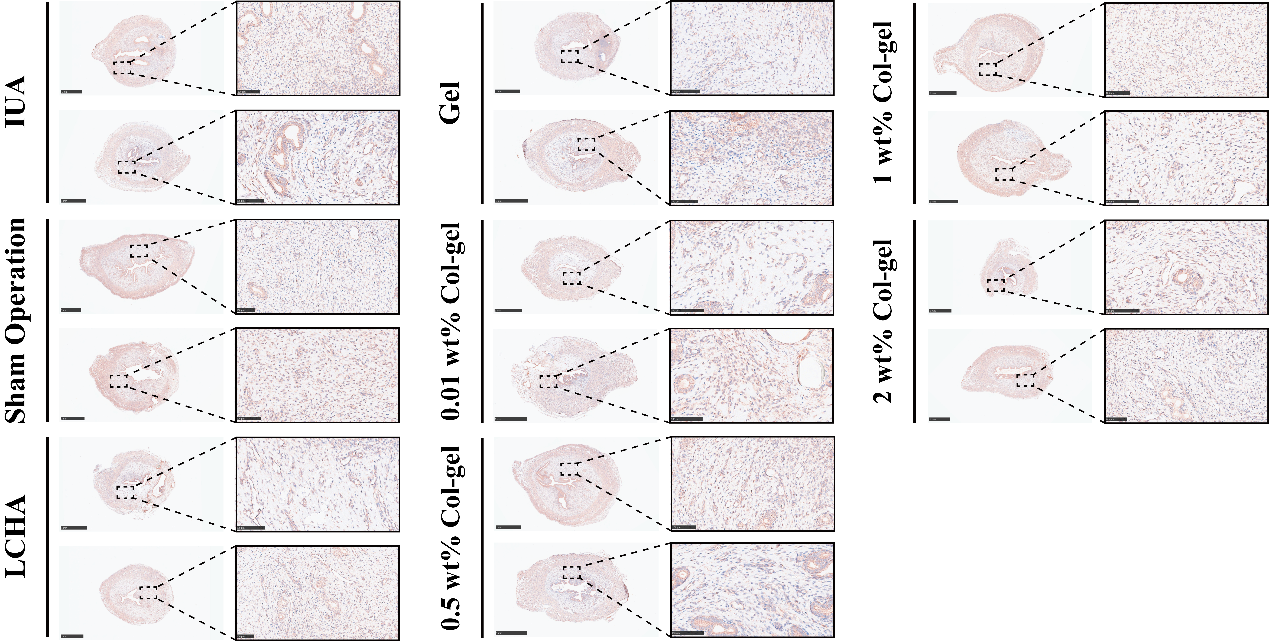


Figure S5. Corresponding TGF-β1 antigen-antibody reaction. Scale bar: 1 mm, 100 µm.


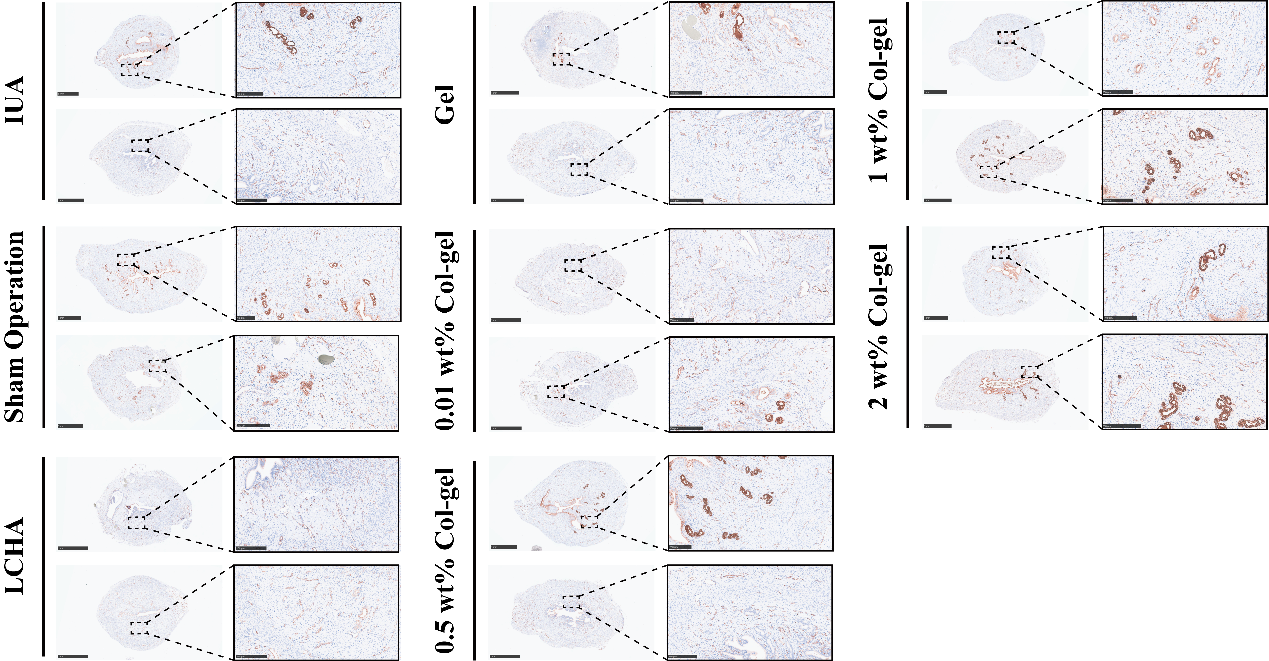


Figure S6. Corresponding CD31 antigen-antibody reaction. Scale bar: 1 mm, 100 µm.


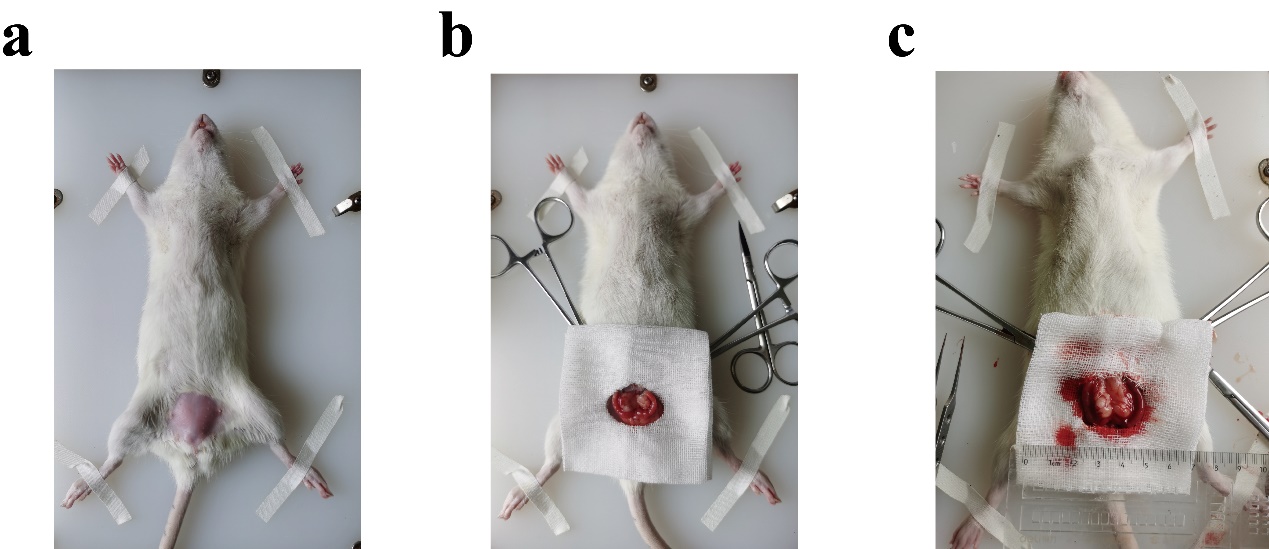


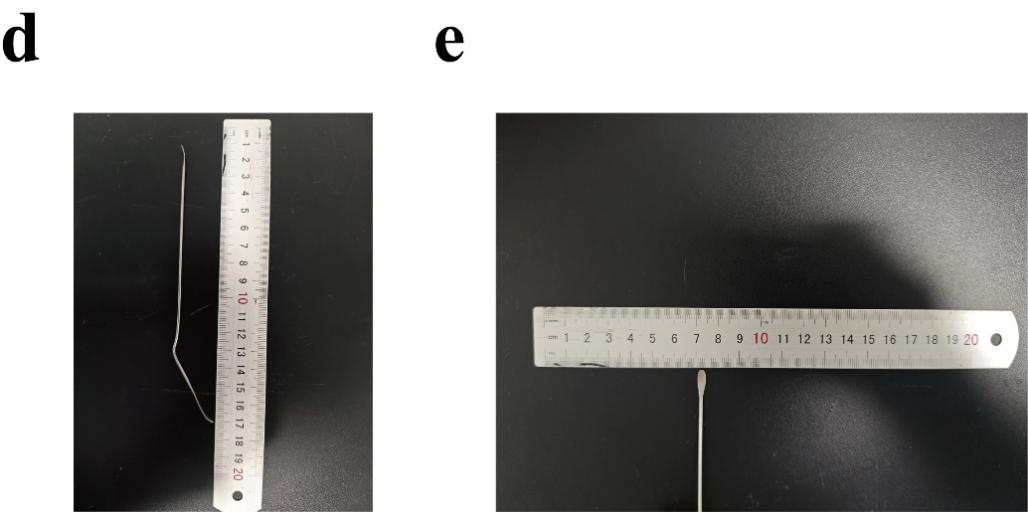


Figure S7. Schematic diagram of IUA rat model establishment. a) Anaesthesia and skin preparation. b) Pick out both sides of the uterus and scrape the uterus with a medicated spoon. c) Redness and swelling of the uterus bilaterally after scraping injury. d and e) Pictures of curettage tools.


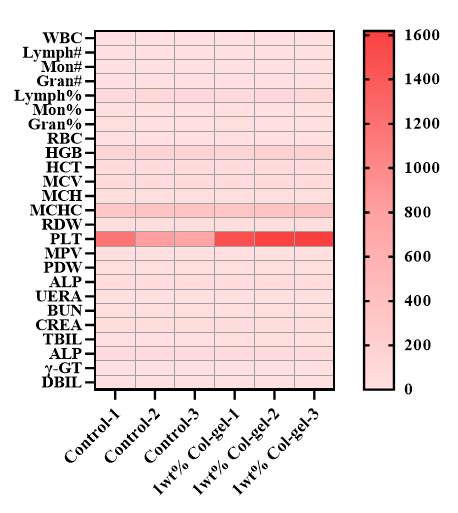


Figure S8. Heatmaps showing indicators of hematology, liver injury (ALB and ALP) and kidney injury (UREA/BUN and CREA) in different groups.
